# Diversity and Antibiotic Susceptibility of *Acinetobacter* Strains From Milk Powder Produced in Germany

**DOI:** 10.3389/fmicb.2018.00536

**Published:** 2018-03-27

**Authors:** Gyu-Sung Cho, Bo Li, André Rostalsky, Gregor Fiedler, Niels Rösch, Etinosa Igbinosa, Jan Kabisch, Wilhelm Bockelmann, Philipp Hammer, Geert Huys, Charles M. A. P. Franz

**Affiliations:** ^1^Department of Microbiology and Biotechnology, Max Rubner-Institut, Kiel, Germany; ^2^Department of Microbiology, Faculty of Life Science, University of Benin, Benin City, Nigeria; ^3^Department of Safety and Quality of Milk and Fish, Max Rubner-Institut, Kiel, Germany; ^4^Laboratory of Microbiology, Ghent University, Ghent, Belgium

**Keywords:** *Acinetobacter*, antibiotic resistance, food, transmission, powdered milk

## Abstract

Forty-seven *Acinetobacter* spp. isolates from milk powder obtained from a powdered milk producer in Germany were investigated for their antibiotic resistance susceptibilities, in order to assess whether strains from food harbor multiple antibiotic resistances and whether the food route is important for dissemination of resistance genes. The strains were identified by 16S rRNA and *rpo*B gene sequencing, as well as by whole genome sequencing of selected isolates and their *in silico* DNA-DNA hybridization (DDH). Furthermore, they were genotyped by rep-PCR together with reference strains of pan-European groups I, II, and III strains of *Acinetobacter baumannii*. Of the 47 strains, 42 were identified as *A. baumannii*, 4 as *Acinetobacter Pittii*, and 1 as *Acinetobacter calcoaceticus* based on 16S rRNA gene sequencing. *In silico* DDH with the genome sequence data of selected strains and *rpo*B gene sequencing data suggested that the five non-*A. baumannii* strains all belonged to *A. pittii*, suggesting that the *rpo*B gene is more reliable than the 16S rRNA gene for species level identification in this genus. Rep-PCR genotyping of the *A. baumannii* strains showed that these could be grouped into four groups, and that some strains clustered together with reference strains of pan-European clinical group II and III strains. All strains in this study were intrinsically resistant toward chloramphenicol and oxacillin, but susceptible toward tetracycline, tobramycin, erythromycin, and ciprofloxacin. For cefotaxime, 43 strains (91.5%) were intermediate and 3 strains (6.4%) resistant, while 3 (6.4%) and 21 (44.7%) strains exhibited resistance to cefepime and streptomycin, respectively. Forty-six (97.9%) strains were susceptible to amikacin and ampicillin-sulbactam. Therefore, the strains in this study were generally not resistant to the clinically relevant antibiotics, especially tobramycin, ciprofloxacin, cefepime, and meropenem, suggesting that the food route probably poses only a low risk for multidrug resistant *Acinetobacter* strains or resistance genes.

## Introduction

Species of the genus *Acinetobacter* (*A*.) belong to the γ-Proteobacteria, and group in the order *Pseudomonadales* and family *Moraxellaceae* (Bouvet and Jeanjean, 1989). Historically, the description of the different species of the genus *Acinetobacter* has been problematic (Krizova et al., 2015). There are currently 42 validly published species names, which include at least one pair of synonyms (Touchon et al., 2014; Krizova et al., 2015). Previous studies have also proposed several “genomic groups” or “genospecies” (Touchon et al., 2014), which have not yet gained formal recognition in nomenclature (Krizova et al., 2015). The species *Acinetobacter baumannii, Acinetobacter Nosocomialis*, and *Acinetobacter pittii* are of clinical importance, as bacteria from these species have been isolated from a number of human infections and hospital outbreaks (Visca et al., 2011). In routine diagnostics, however, it is difficult to differentiate accurately between *A. baumannii, A. pittii, A. nosocomialis*, and *Acinetobacter calcoaceticus* strains, as these are highly similar with respect to their phenotypic and biochemical properties. Furthermore, these strains show close relationship in DNA-DNA hybridization (DDH) studies. Therefore, these have commonly been grouped together in the so-called *A. calcoaceticus*–*A. baumannii* complex (Sheng et al., 2011).

*Acinetobacter baumannii* is one of the six most important multi-drug resistant microorganisms in hospitals world-wide (Talbot et al., 2006; Antunes et al., 2014) and in 2005 was estimated to cause 2–10% of all Gram-negative bacterial hospital infections (Antunes et al., 2014). Multidrug-resistant *A. baumannii* are responsible for severe hospital-acquired infections (bloodstream, skin, soft tissue, wound, urinary tract, pulmonary, ventilator-associated pneumonia, and device-related infections) and are frequently isolated from patients hospitalized in intensive care units (Antunes et al., 2014; Potron et al., 2015). The emergence of *A. baumannii* as a major nosocomial pathogen has been attributed not only to specific characteristics of the organism such as multidrug resistance and its survival for long periods on surfaces and medical equipment, but also to human factors such as host health status and its association with person-to-person transmission (Medell et al., 2012; Tuon et al., 2015). The increase in antibiotic resistance of *A. baumannii* has consequently reduced therapeutic options for inhibition of this pathogen (Dijkshoorn et al., 2007). The bacterium is known to survive for extended length in the nosocomial setting and can even cause recurrent outbreaks of e.g., pneumonia, especially in emergency and intensive care units (Peleg et al., 2008; Ahmed et al., 2015). In Germany, a serious nosocomial outbreak with *A. baumannii* occurred during December 2014/January 2015 in the University Clinic Schleswig Holstein [Universitätsklinikum Schleswig-Holstein (UKSH)] in the city of Kiel, when a multiresistant *A. baumannii* strain infected 31 patients and resulted in several deaths, leading to a temporary closure of the intensive care unit. This strain was resistant toward penicillins, cephalosporins, carbapenems, and fluoroquinolones (Martins et al., 2014).

Antibiotics are being used for therapeutical purposes in livestock production on a relative large scale, and this has been linked to the emergence and spread of resistant bacteria from animals and animal-derived foods to people (Hamouda et al., 2011). For *Acinetobacter*, however, a previous study (Hamouda et al., 2011) showed that *A. baumannii* strains from a Scottish abattoir did not possess epidemiological characteristics that are similar to strains isolated from clinics, and the authors concluded that the *A. baumannii* isolates from animals investigated in that study did not appear to have evolved to strains causing hospital-acquired infections. *Acinetobacter baumannii* is also known to occur in animal-derived products such as bulk tank milk (Straley et al., 2006; Gurung et al., 2013; Tamang et al., 2014) and in infant milk formula (Wang et al., 2009; Miled et al., 2010; Araújo et al., 2015; Juma et al., 2016). In a report on a meeting on *Cronobacter sakazakii* and *Salmonella* in powdered infant formula, the FAO/WHO classified *A. baumanii* as an emerging pathogen “Category-B” microorganisms—“causality plausible, but not yet demonstrated” (FAO/WHO, 2006).

*Acinetobacter* spp. isolates in this study stemmed from a powdered milk production facility, which employs the roller-drying method for milk powder production. The manufacturer performs a permanent microbiological trend analysis (MTA) which primarily aims to monitor the presence of *Enterobacteriaceae* in the final product. In a joint research project, the Max Rubner-Institut received violet red bile dextrose agar plates (VRBD) with bacterial growth from the producer's MTA samples for further characterization. This study aimed to determine whether foodborne *Acinetobacter* isolates in dried milk possessed similar antibiotic resistances as the clinical strains, and to assess whether *Acinetobacter* strains can function as potential reservoirs of antibiotic resistance along the food transmission route and my later become problematic in a hospital setting when hosts are immunocompromised.

## Materials and methods

### Strain isolation and culturing

All *Acinetobacter* spp. food isolates used in this study originated from dried milk produced via rotating drum drying by a company in Germany. Dried milk samples were taken directly from the end of the production line. Five samples of 10 g each were taken daily at various time points. Microbiological analysis for enterobacteria was done after enrichment in double buffered peptone water [peptone water 20.0 g L^−l^ (Oxoid, Wesel, Germany), 3.5 g L^−l^ Na_2_HPO_4_ (Merck), 1.5 g L^−l^ KH_2_PO_4_, Merck] by plating out onto VRBD (Becton Dickinson, Heidelberg, Germany) agar medium in the laboratory of the quality control department of the producing company. Plates were surface spread in order to isolate colonies and incubated at 30°C for 24 h aerobically. VRBD plates showing bacterial growth were transferred to the Max Rubner-Institut for further isolation, characterization, and identification of the enterobacteria strains. For this, morphologically different colonies were selected for isolation (random selection). This resulted in 47 isolates from different plates, which were phenotypically characterized by Gram-staining and were presumptively identified as *Acinetobacter* spp. by API 20E (BioMérieux, Nürtingen, Germany), to occur amongst other enterobacteria. These strains were further characterized by genotypic fingerprinting using rep-PCR fingerprinting. In addition, five clinical *A. baumannii* strains obtained from the BCCM/LMG Bacteria Collection (Ghent University, Ghent, Belgium), were used as reference strains of the three pan-European genomic subgroups of *A. baumannii* (I, LMG 10543; II, LMG 22458, LMG 10541; III, LMG 22452, LMG 22863) (Huys et al., 2005a). All *Acinetobacter* strains were routinely cultured using LB medium (VWR, Darmstadt, Germany) at 37°C.

### Strain genotyping by rep-PCR

For rep-PCR fingerprinting, total genomic DNA was isolated with ZR Fungal/Bacterial DNA MiniPrep™ kit (Zymo Research, Freiburg, Germany) following the manufacturer's instruction, and the final DNA concentration was measured with a Nanodrop (Peqlab, Erlangen, Germany) and adjusted to 10 ng μL^−l^. The primer used was the (GTG)_5_ (5′-GTG GTG GTG GTG GTG-3′) primer (Huys et al., 2005a) and amplification conditions were as described previously by Huys et al. (2005a). PCR products were separated by electrophoresis on a 1.8% agarose gel using 1 X TBE (Tris-Borate-EDTA) buffer. Gels were stained in GelRed (Bio Trend, Cologne, Germany) and photographed on an UV transilluminator. Photo-positives were digitized, after which scanned images were normalized and analyzed using the Bionumerics (v. 7.1) software package (Applied Maths, Sint-Martens-Latem, Belgium). Clustering analysis of the rep-PCR fingerprints was performed by means of the Pearson product-moment correlation coefficient (*r*) and the unweighted pair-group method using arithmetic averages clustering algorithm (UPGMA) using Bionumerics (v. 7.1).

### Strain identification

Strains were identified by sequencing the 16S rRNA and *rpo*B genes. For this, the DNA of the strains was isolated with ZR Fungal/Bacterial DNA MiniPrep™ kit (Zymo Research) described above. The 16S rRNA and the *rpo*B genes were amplified as described previously (Kostinek et al., 2005; Higgins et al., 2010b). The PCR products were cleaned using the PCR cleaning kit from Qiagen (Hilden, Germany) and were sequenced bi-directionally at GATC Biotech (Cologne, Germany). Multiple aligned sequences were clustered and similarities were calculated with the UPGMA algorithm in Bionumerics v. 7.1. In addition, 12 strains, representative of different 16S rRNA gene sequence clusters, were selected for whole genome sequencing in order to confirm the species identities.

### Antibiotic resistance testing

Antibiotic resistance testing was done by the Kirby-Bauer disc diffusion method (CLSI, 2015; EUCAST, 2016). For disc diffusion assays, isolates were incubated in Mueller Hinton broth (Merck, Darmstadt) at 37°C overnight and the turbidity of the fresh culture was adjusted to 0.5 McFarland scale. A 100 μL of adjusted, fresh overnight culture was plated out onto Mueller Hinton agar (Merck). After drying the plates, antibiotic discs (Oxoid, Wesel, Germany) were applied onto the surface using a dispenser. The antibiotic discs used in this study included ampicillin (AMP, 10 μg), chloramphenicol (C, 30 μg), ciprofloxacin (CIP, 5 μg), cefepime (FEP, 30 μg), cefotaxime (CTX, 5 μg), erythromycin (E, 15 μg), meropenem (MEM, 10 μg), oxacillin (OX, 5 μg), streptomycin (S, 10 μg), tetracycline (TET, 30 μg), and tobramycin (TOB, 10 μg). All plates were incubated at 35°C for 18 h as described in the CLSI (CLSI, 2015) and EUCAST (EUCAST, 2016) guidelines. After incubation, the diameter of the inhibition zone was measured and the isolates were grouped into the categories susceptible, intermediate or resistant, based on the diameter of the inhibition zone for the respective antibiotic, according to the CLSI reference (for oxacillin, streptomycin, tetracycline, chloramphenicol, ampicillin, cefotaxime, cefepime, and erythromycin) or EUCAST reference (for ciprofloxacin, meropenem, and tobramycin) for *Acinetobacter* spp. The inhibition zone diameters used for classifying the microorganism's antibiotic susceptibilities are shown in Table 1. All antibiotic resistance determinations were done in duplicate.

**Table 1:** Antibiotic susceptibility of 47 *Acinetobacter* strains using the disc diffusion test.

**Antibiotic**	**Disc diffusion test**		
	**Susceptible strains (x/47;%)**	**Intermediate resistant strains (x/47;%)**	**Resistant strains (x/47;%)**
CTX	339/08/885 **(1/47; 2.1%)**	**(44/47; 91.5%)**	10/1255, 12/1258, 73/7515 **(3/47; 6.4%)**
C	None	None	**(47/47; 100%)**
TET	All **(47/47; 100%)**	None	None
TOB	All **(47/47; 100%)**	None	None
CIP	All **(47/47; 100%)**	None	None
E	**(47/47; 100%)**	None	None
S	3/700, 8/889, 10/1255, 12/1258, 25/2244, 43/18, 64/5920, 66/6106, 70/6114, 73/7515, 76/7688, 82/8590, 132/2279, 133/2516, 156/4802, 185/7652, 224/9988, 221/9981, 229/401, 249/1482, 260/2529, 339/08/855, 340/08/856, 381/08/7035, 333/08/640 **(27/47; 55.3%)**	none	15/1623, 16/1775, 20/1954, 44/4, 81/8327, 99/648, 102/871, 109/1450, 116/1882, 127/2080, 147/4208, 242/1259, 258/2368, 286/07/5411, 298/07/6825, 300/07/8311, 305/07/8677, 313/07/9003, 315/07/9006, 318/07/9217, 359/08/2546 **(21/47; 44.7%)**
MEM	**(47/47; 97.9%)**	16/1775 **(1/47; 2.1%)**	None
AK	**(47/47; 97.9%)**	339/08/885 **(1/47; 2.1%)**	None
SAM	**(47/47; 97.9%)**	82/8590 **(1/47; 2.1%)**	None
FEP	**(45/47; 93.8%)**	None	12/1258, 82/8590, 99648 (3/47; 6.4%)

*Susceptibility toward the antibiotics was classified according to the CLSI (2015) and EUCAST (2016) guidelines. C, chloramphenicol; S, streptomycin; TET, tetracycline; E, erythromycin; FEP, cefepime; CTX, cefotaxim; AK, amikacin; SAM, ampicillin-sulbactam; CIP, ciprofloxacin; TOB, tobramycin; MEM, meropenem. Bold values indicate the number of resistant strains out of total 47, followed by the corresponding the percentage resistant*.

### PCR screening for antibiotic resistance genes

For screening of antibiotic resistance genes, the total genomic DNA of all strains was isolated using the ZR Fungal/Bacterial DNA MiniPrep™ kit. Primers and multiplex PCR conditions for amplification of carbapenem-hydrolyzing, class D β-lactamases (CHDLs), blaOXA-23, *bla*OXA-24, *bla*OXA-51, and *bla*OXA-58, were as described previously (Ma et al., 2015). The primers and amplification conditions used to amplify the *cat*(I) and *tet*(A) genes were also previously described (Sianglum et al., 2009; Vilacoba et al., 2013).

### Whole genome sequencing

The genomic DNA of *Acinetobacter* strains was isolated using the peqGOLD bacterial DNA kit (Peqlab, Erlangen, Germany). The sequencing library was prepared with an Illumina Nextera XT library prep kit (Illumina, San Diego, USA) and run on the MiSeq platform present in our MRI laboratory with 2 × 300 paired-ends. All obtained paired-end and unpaired-end reads were assembled *de novo* using SPAdes version 3.10.1 (Bankevich et al., 2012). The assembled contigs were utilized for *in silico* detection and typing of acquired antibiotic resistance genes, multi locus sequencing typing (MLST), as well as plasmid detection and identification using the ResFinder database (Zankari, 2014), PubMLST (Bartual et al., 2005), and PlasmidFinder (Carattoli et al., 2014) pipelines, respectively. In addition, the contigs related to plasmid sequences were extracted from whole-genome sequence data and were applied to BacMet pipeline (Pal et al., 2014) to identify antibacterial biocide and metal resistance genes. The draft genomes of three isolates clustering together with the *A. pittii* and *A. calcoaceticus* type strains in 16S rRNA and *rpo*B gene sequence analyzes were also used for *in silico* DDH together with the sequenced *A. pittii* PHEA-2 and *A. calcoaceticus* NCTC 7364 reference strains, using the web-based program at http://ggdc.dsmz.de. Whole genome comparisons of the nine sequenced *A. baumannii* isolates and the *A. baumannii* AB030 clinical reference strain were done using the Blast Ring Image Generator (BRIG) (Alikhan et al., 2011). The strains were compared especially for the localization of antibiotic resistance genes and selected virulence genes.

## Results

### Whole genome sequencing

**Figure 1:**
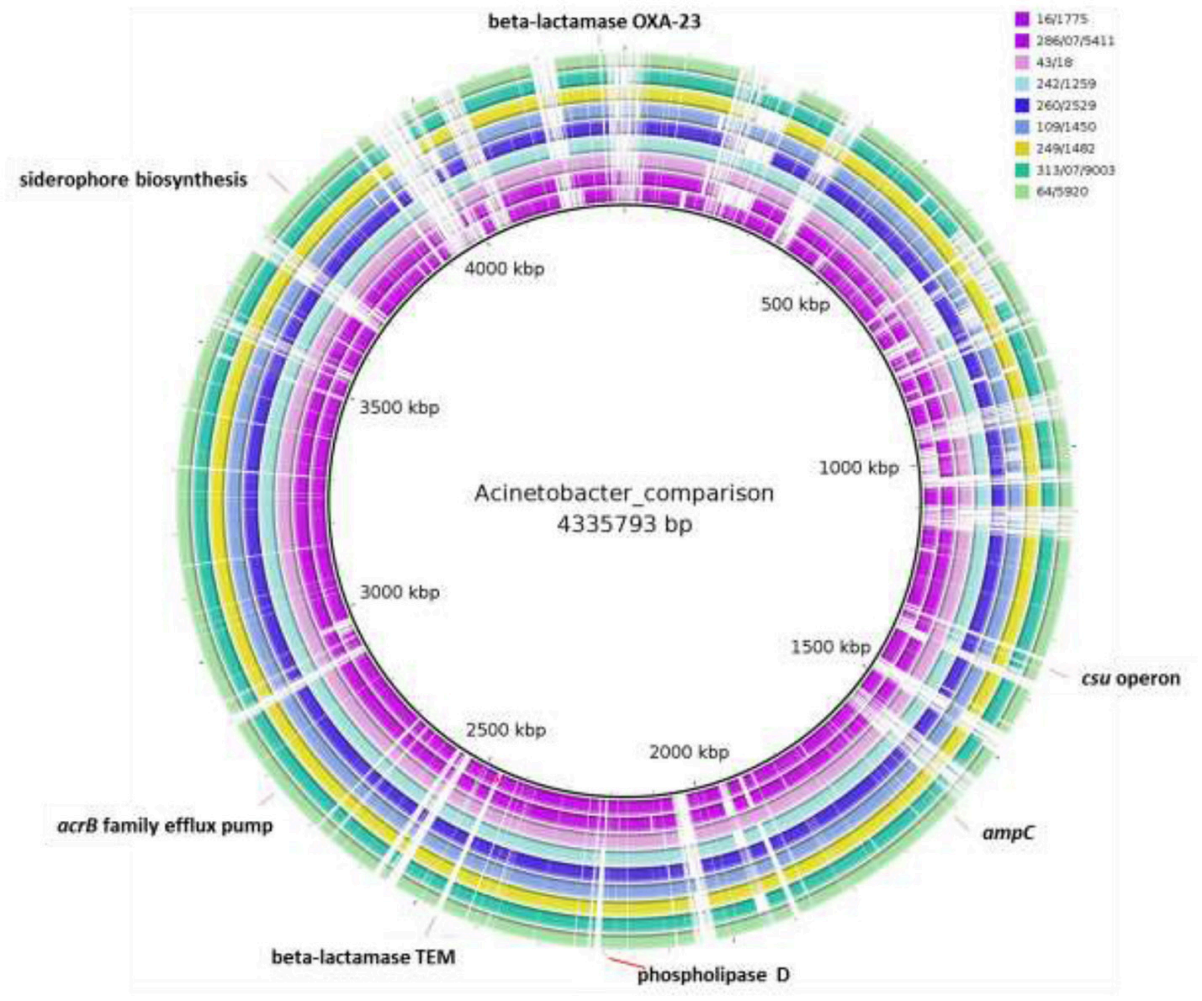
Whole-genome comparisons of food *Acinetobacter baumannii* isolates considered in this study and the *A. baumannii* AB030 clinical strain. From outer to inner ring: *A. baumannii* 64/5920, *A. baumannii* 43/18, *A. baumannii* 313/07/9003, *A. baumannii* 260/2529, *A. baumannii* 286/07/5411, *A. baumannii* 249/1482, *A. baumannii* 242/1259, *A. baumannii* 16/1775, *A. baumannii* 109/1450, reference genome: *A. baumannii* AB030. The loci of selected virulence genes, antibiotic resistance genes are indicated. The gaps shown indicate where the sequence data from isolates differ from the clinical strain and in these gaps the position of antibiotic resistance genes present in the clinical strain but not in the isolates (e.g., TEM-1 and OXA-23) are shown.

The paired-end and unpaired-end sequence reads of the 12 selected *Acinetobacter* strains (9 *A. baumannii* and 3 *A. pittii* strains) were *de novo* assembled and generated into contigs. The N50 values ranged from 31,249 to 188,943 bp and the genome sizes of these strains were from 3.72 to 4.36 Mbp (Table 2). These genome sizes compared well to the range of sizes of between 3.70 to 4.85 Mbp reported by Wallace et al. (2016). Genes encoding virulence factors such as the genes for siderophore production, the *csu* operon involved in biofilm production and phospholipase D genes, as well as genes associated with efflux pump mediated antibiotic resistance such as the AcrB family efflux pump were indicated using BRIG (Figure 1). The plasmid sequences from assembled genome sequences, as well as resistance genes to metals and antibacterial biocides were identified by the BacMet web-based program and are shown in Table 3. The presence of specific virulence genes present on the chromosome of the whole genome sequenced isolates are also shown in Table 3.

**Table 2:** Results of *de novo* genome assembly with SPAdes pipeline and *in silico* detection of antibiotic resistant genes and MLST of *Acinetobacter baumannii/Acinetobacter pittii*.

**Sample**	**Genome size (bp)**	**Contigs no**.	**N50**	**No. of RNAs**	**No. of cording sequences**	**GC content (mol%)**	**Antibiotic resistance genes**	**MLSTs**
258/2368	4,054,962	101	98,038	70	3,830	38.8	*bla*ADC-25-like; streptomycin 3″-O-adenylyltransferase	
81/8327	4,308,663	298	88,604	70	4,096	38.8	*bla*ADC-25-like; streptomycin 3″-O-adenylyltransferase	
290/07/6825	4,024,466	157	53,670	73	3,781	38.8	*bla*ADC-25-like; streptomycin 3″-O-adenylyltransferase	
16/1775	3,774,409	218	38,174	70	3,648	39.0	*bla*ADC-25-like,*bla*OXA-69-like; streptomycin 3″-O-adenylyltransferase	Unknown ST
286/07/5411	3,694,590	288	27,470	61	3,497	39.2	*bla*ADC-25-like,*bla*OXA-180-like; streptomycin 3″-O-adenylyltransferase	
43/18	3,877,281	175	60,476	66	3,566	39.1	*bla*ADC-25-like,*bla*OXA-69-like; streptomycin 3″-O-adenylyltransferase	Unknown ST
242/1259	3,896,179	126	79,304	65	3,677	39.0	*bla*ADC-25-like,*bla*OXA-64; streptomycin 3″-O-adenylyltransferase	Unknown ST
260/2529	4,058,737	128	67,655	69	3,782	39.0	*bla*ADC-25-like,*bla*OXA-91; streptomycin 3″-O-adenylyltransferase	ST-613
109/1450	3,918,108	261	33,652	73	3,735	38.9	*bla*ADC-25-like,*bla*OXA-69-like; streptomycin 3″-O-adenylyltransferase	ST-427
249/1482	4,067,831	63	122,702	69	3,803	39.0	*bla*ADC-25-like,*bla*OXA-91; streptomycin 3″-O-adenylyltransferase	ST-613
313/07/9003	3,895,889	100	77,208	65	3,673	38.9	*bla*ADC-25-like,*bla*OXA-64; streptomycin 3″-O-adenylyltransferase	Unknown ST
64/5920	3,879,717	98	91,028	65	3,577	38.8	*bla*ADC-25-like,*bla*OXA-91; streptomycin 3″-O-adenylyltransferase	ST-613

**Table 3:** Plasmid sequence identity, metal resistance genes, and virulence factors identified in *Acinetobacter baumannii* strains by genome sequencing in this study.

**Strain**	**Plasmid (% identity)^a^**	**Metal resistance gene^b^**	**Virulence factor^c^**
16/1775			OmpA, siderophore, phospholipase D, CsuA, OmpR/EnvZ
286/07/5411	Col440II (86.26%)	Cobalt-zinc-cadmium resistance protein *czc*A	OmpA, phospholipase D, csuA, ompR/EnvZ
43/18	Col440I (90.09%) Col440II (86.73%)	Heavy metal translocating P-type ATPase	OmpA, siderophore, phospholipase D, CsuA, OmpR/EnvZ
242/1259			OmpA, siderophore, phospholipase D, OmpR/EnvZ
260/2529			OmpA, siderophore, phospholipase D, CsuA, ompR/EnvZ
109/1450			OmpA, siderophore, phospholipase D, CsuA, ompR/EnvZ
249/1482	ColRNAI (90.43%)	Cation efflux system protein *cus*A	OmpA, siderophore, phospholipase D, CsuA, ompR/EnvZ
313/07/9003			OmpA, siderophore, phospholipase D, CsuA, ompR/EnvZ
64/5920			OmpA, siderophore, phospholipase D, CsuA, ompR/EnvZ

a *Identity of genome sequence contigs with reported plasmid sequences was determined with PlasmidFinder*.

b *Metal resistance gene: the first Blastx hit from BacMet database*.

c *OmpA: involved in epithelial cell invasion and apoptosis; siderophores: iron acquisition, survival in human serum; CusA: pili involved in biofilm formation and maintenance; phospholipase C/D: survival in human serum and epithelial cell invasion; OmpR/EnvZ: killing of host cells*.

### Strain typing and identification

**Figure 2:**
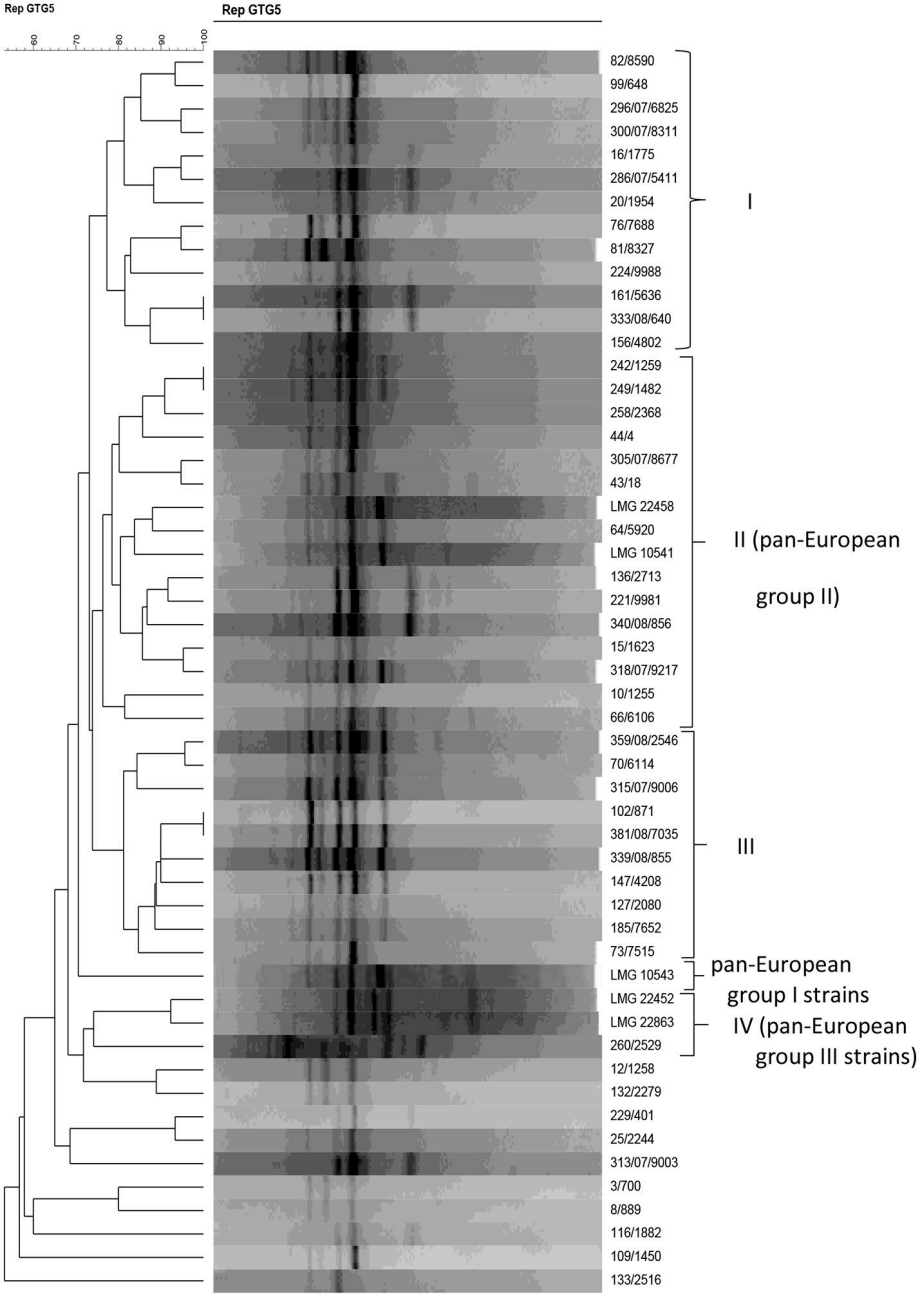
Dendrogram obtained by UPGMA using Pearson correlation coefficient *r* of digitized (GTG)_5_-PCR fingerprints of *Acinetobacter* isolates from powdered milk and reference strains.

*Acinetobacter* strains were characterized genotypically by rep-PCR analysis with primer (GTG)_5_, together with the five *A. baumannii* reference strains previously shown to belong to one of the three *A. baumannii* clonal complexes that can be discriminated by (GTG)_5_-PCR (Huys et al., 2005a). rep-PCR fingerprinting grouped the strains in four groups with a similarity *r* of ~ 75% or more (Figure 2). Thirteen strains clustered in group I at *r* = 77.3% and 14 isolates clustered in group II (*r* = 76.3%) together with the *A. baumannii* reference strains LMG 10541 and LMG 22458 which belong to pan-European group II (Huys et al., 2005a,b). Group III contained 10 strains which clustered at *r* = 81.2%, and one strain in group IV clustered together with the pan-European group III reference strains at *r* = 74.2%. Strain LMG 10543, representative of a pan-European clone group I, clustered apart from the other strains included in this study. The remaining 10 strains (i.e., 12/1258, 132/2279, 229/401, 25/2244, 313/07/9003, 3/700, 8/889, 116/1882, 109/1450, and 133/2516) clustered apart from all other strains.

**Figure 3:**
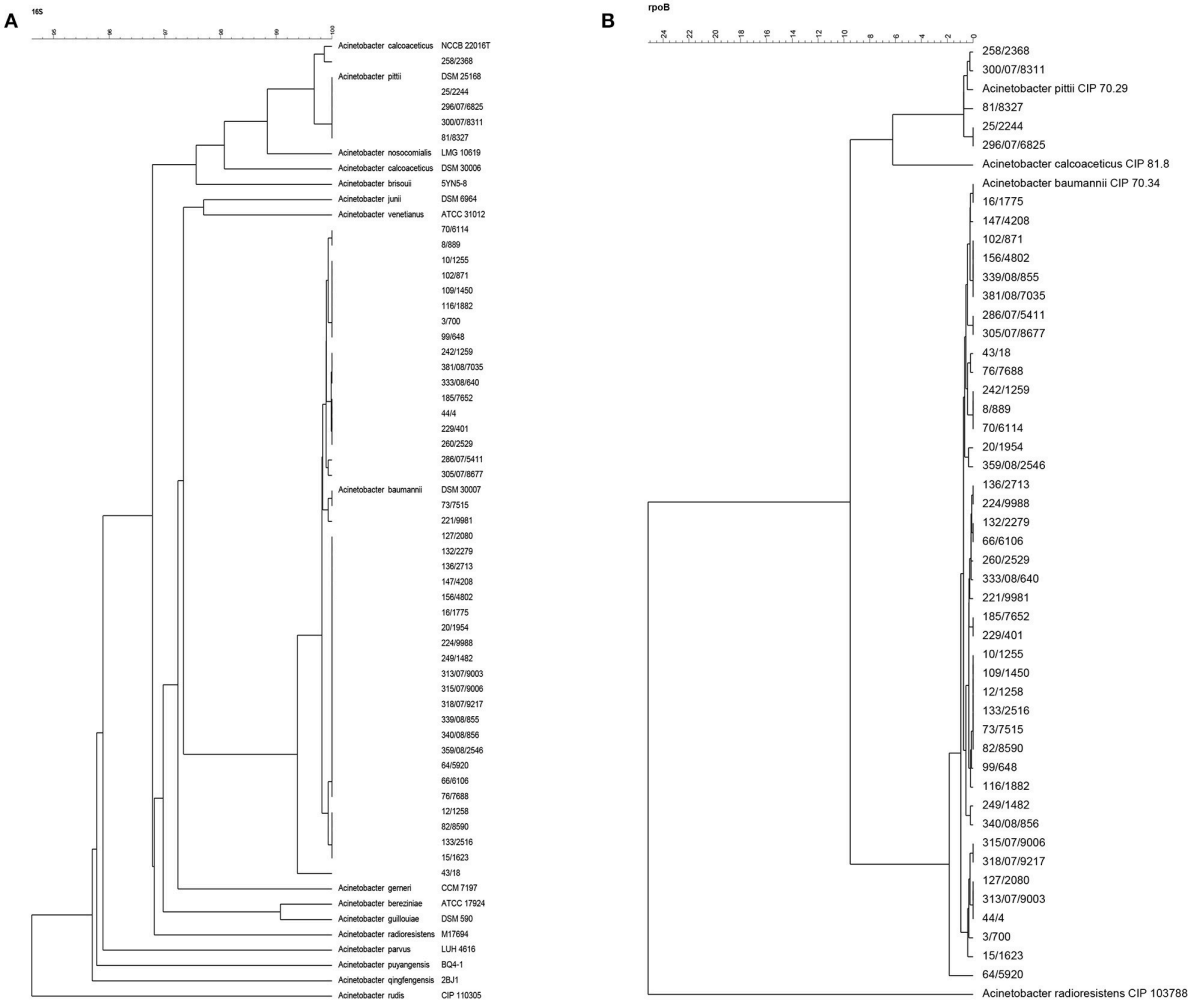
Phylogenetic tree based on multiple aligned sequences of *Acinetobacter* isolates from powdered milk. **(A)** 16S rRNA gene sequence analysis; **(B)** *rpo*B gene sequence analysis.

All strains investigated with rep-PCR fingerprinting were also identified by 16S rRNA (Figure 3A) and *rpo*B (Figure 3B) gene sequencing. Forty-two of the 16S rRNA gene sequences of strains clustered together with the corresponding sequence of the *A. baumannii* type strain DSM 30007^T^ at a high similarity of 99.4% (Figure 3A). Similar to 16S rRNA gene cluster, the *rpo*B gene sequences of the same 42 *A. baumannii* strains also clustered together with type strain (*A. baumannii* CIP 70.34^T^ = *A. baumannii* DSM 30007^T^) at *r* = 98.2% (Figure 3B). Given the consensus between both sequenced genes, these strains were all confirmed as members of *A. baumannii*. Four of the remaining strains, i.e., 25/2244, 296/07/6825, 300/07/8311, and 81/8327, were characterized as *A. pittii* strains based on their 16S rRNA gene sequences which clustered at 100% similarity together with the corresponding gene sequence of the *A. pittii* DSM 25618^T^ type strain. As for the 16S rRNA gene clustering results, these four strains grouped together with the *A. pittii* type strain (*A. pittii* CIP 70.29^T^ = *A. pittii* DSM 25618^T^) at 99.3% similarity also in the *rpo*B gene cluster. In 16S rRNA gene analysis, strain 258/2368 clustered closely at *r* = 99.9% with the *A. calcoaceticus* type strain NCCB 22016^T^ (Figure 3A), whereas based on *rpo*B gene sequencing this strain did not cluster with the *A. calcoaceticus* type strain but with the A. *pittii* type strain DSM 25168^T^ (= CIP 70.29) (Figure 3B). For this reason, the genome sequences of the three strains 258/2368, 81/8327, and 296/07/6825 were used for *in silico* DDH together with reference strains *A. calcoaceticus* NCTC 7364 and *A. pittii* PHEA-2. The three strains showed DDH-values of ~69% when hybridized with *A. pittii* PHEA-2, and ~35% when hybridized with *A. calcoaceticus* NCTC 7364. This showed that the three strains 258/2368, 296/07/6825, and 81/8327, clustering with *A*. *calcoaceticus* CIP 81.8^T^ and *A. pittii* CIP 70.29^T^ type strains in 16S rRNA and *rpo*B gene sequence analyzes (Figures 3A,B), were in fact all *A. pittii* strains, as was suggested by the clustering analysis based on the *rpo*B gene sequences (Figure 3B). As the strains 300/07/8311 and 25/2244 also grouped with these sequenced strains and the *A. pittii* type strain in both 16S rRNA gene and *rpo*B gene clustering, it can de deduced that these two strains can also be identified as *A. pittii*.

Using rep-PCR typing, the *A. pittii* strains 258/2368 and 81/8327, 296/07/6825, and 300/07/8311 occurred in two clusters (i.e., rep-PCR groups I and II), respectively, together with most of the *A. baumannii* strains. The *A. pittii* strain 25/2244 did not cluster into any of the rep-PCR groups. This implies that rep-PCR using the (GTG)_5_ primer cannot be used for successful species delineation of *Acinetobacter* isolates from food.

### Antibiotic resistance

**Figure 4:**
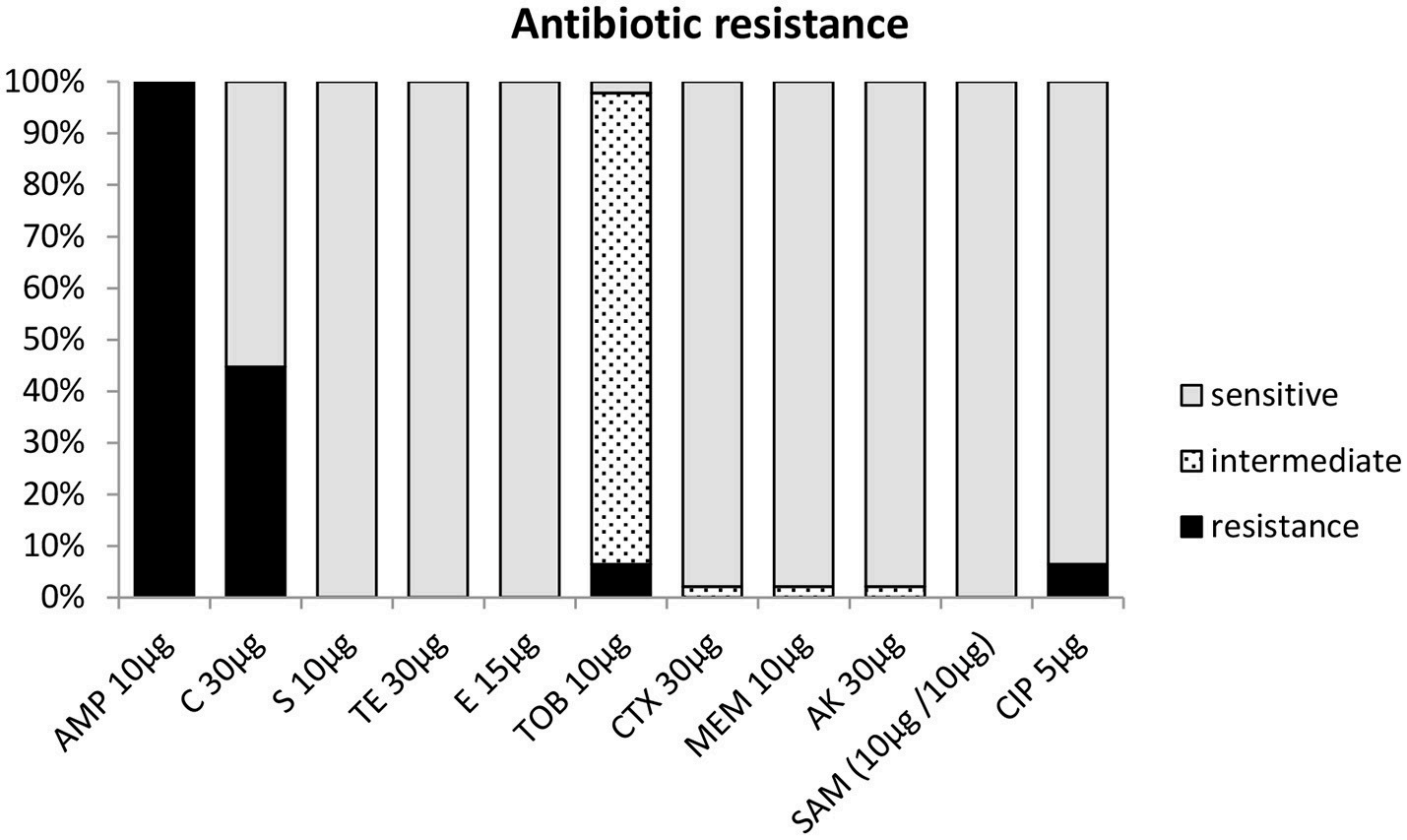
Antibiotic resistance profile of *Acinetobacter* strains based on inhibition zone diameter (mm) according to CLSI (2015) C, chloramphenicol; S, streptomycin; TET, tetracycline; E, erythromycin; FEP, cefepime; CTX, cefotaxim; AK, amikacin; SAM, ampicillin-sulbactam; and EUCAST (2016) CIP, ciprofloxacin; TOB, tobramycin; MEM, meropenem.

All 47 strains were screened for antibiotic resistance using the disc diffusion method. Depending on the presence and size (diameter) of an inhibition zone, strains tested by this method were classified as either being susceptible, intermediate, or resistant toward an antibiotic (Table 1). The antibiotic resistance profiles of the strains from milk powder in this study are shown in Figure 3B and Table 1. All strains were resistant toward chloramphenicol (30 μg) and oxacillin (5 μg) (Table 1), but were susceptible to tetracycline, ciprofloxacin, tobramycin, and erythromycin. Testing against meropenem, amikacin, and ampicillin-sulbactam showed that 46 (97.9%) strains were susceptible to these antibiotics, while only one (2.1%) strain showed an intermediate resistance to each of these antibiotics (Figure 4, Table 1). Forty-three strains (93.8%) were susceptible toward cefepime. About half of the strains were resistant toward streptomycin (44.7%). For cefotaxime, only 3 strains (6.4%) were resistant, while 43 (91.5%) were intermediate and a single strain (2.1%) was susceptible (Figure 4, Table 1).

Conventional PCR amplification of selected antibiotic resistance genes such as *bla*OXA-23, *bla*OXA-24, *bla*OXA-51, and *bla*OXA-58 involved with imipenem and meropenem resistance were carried out. All 42 strains identified as *A. baumannii* were PCR-positive for *bla*OXA-51 family genes only. Of these, 34 strains gave a PCR product that was sequenced and showed over 99% identity to *bla*OXA-51 gene sequences in Genbank (results not shown). Four strains (242/1259, 44/4, 315/07/9006, and 242/1259) yielded larger size PCR products than the expected *bla*OXA-51 conventional PCR fragment (data not shown). These products were sequenced and they showed over 99% sequence identity with the *bla*OXA-530 gene, which also belongs to the *bla*OXA-51 family. The remaining four *A. baumannii* strains (249/1482, 43/18, 133/2516, and 132/2279) also gave PCR products of larger size when compared to the *bla*OXA-51 PCR product and were found to correspond to *bla*OXA-91 and *bla*OXA-430 genes of the *bla*OXA-51 family (data not shown). The five *A. pittii* strains were all negative for *bla*OXA-51.

Genome sequencing of the nine *A. baumannii* strains showed that these carried various *bla*-OXA genes which belong to the *bla*OXA-51 family, i.e., *bla*OXA-64, *bla*OXA 69-like, *bla*OXA-91, and *bla*OXA-180-like (Table 2). Furthermore, all strains contained a *bla*ADC-25-like gene, which encodes an intrinsic AmpC beta-lactamase (Boo and Crowley, 2009). Interestingly, genome sequencing of the three strains identified as *A. pittii* showed that these strains only possessed the AmpC beta-lactamase, while no *bla*OXA-51-like family genes could be determined. This was in agreement with the PCR results. A BRIG comparison of the nine *A. baumannii* genomes with a clinical reference strain (*A. baumannii* ABO30 acc no: CP009257.1) showed that regions in which the important antibiotic resistance genes i.e., *bla*OXA23 carbapenem-hydrolyzing class D and *bla*TEM-1 ESBL are located, are absent in the food isolates (Figure 1). A few genome studies have previously identified a common core set of genes, including virulence genes such as phospholipase D and the *csu* operon (Figure 1, Table 3) and a highly variable accessory genome in *A. baumannii*, the latter of which includes many of the acquired antibiotic resistance genes (Chan et al., 2015; Li et al., 2015).

## Discussion

*Acinetobacter* spp. including *A. baumannii* commonly occur in raw bulk tank milk (Straley et al., 2006; Gurung et al., 2013; Tamang et al., 2014), with one investigation reporting up to 7.7% of raw, bulk tank milk samples being positive for species of this genus (Gurung et al., 2013). In the current study, the majority of *Acinetobacter* isolates (89.4%) were identified as *A. baumannii* by 16S rRNA and *rpo*B gene sequencing, while five isolates were identified by 16S rRNA gene, *rpo*B gene, and *in silico* DDH as *A. pittii*. In comparison, the study of Gurung et al. (2013) reported that 57 of 176 (32.4%) *Acinetobacter* isolates from raw bulk tank milk were identified as *A. baumannii*.

In this study, most of the *A. baumannii* strains grouped in one of three (GTG)_5_—PCR fingerprint groups. Huys et al. (2005a,b) showed the value of rep-PCR with (GTG)_5_ primer for identifying *A. baumannii* and for resolving its epidemiologically successful clonal lineages [13]. These included the pan-European clone groups I and II, which were suggested to usually contain either a *tet*(A) or a *tet*(B) gene, respectively, and members of the clonal group III, which were suggested to often contain a *aph*A6 gene conferring resistance to aminoglycosides (Huys et al., 2005a,b). In our study, the clustering analysis of rep-PCR profiles indicated that the *Acinetobact*er isolates from powdered milk contained only strains representative of pan-European clonal lineages II and III (Figure 2). However, the strains investigated were all tetracycline susceptible and were not clinical isolates, as was the case for the strains described for the pan-European clone group strains in the study of Huys et al. (2005a,b). At the species level, rep-PCR with the (GTG)_5_ primer appeared less suitable as a taxonomic tool, given that strains identified as *A. pittii, A. calcoaceticus*, or *A. baumannii* strains using 16S rRNA and *rpo*B gene sequencing did not group in separate rep-PCR clusters. These results indicate also that food isolates do not necessarily group into clonal lineages like clinical isolates do and thus rep-PCR with primer GTG_5_ may not be appropriate for typing of food isolates by this method.

Resistance toward ß-lactam antibiotics is an important part of multidrug resistance in *A. baumannii*, and all four Ambler classes (A, B, C, and D) of ß-lactamase resistance can be found in this microorganism (Lin and Lan, 2014; Potron et al., 2015). All strains in this study from powdered milk were phenotypically oxacillin resistant, which is a well-known trait for *A. baumannii* strains. Resistance to this antibiotic is most often the result of class D β-lactamases or oxacillinases which is generally chromosomally encoded. Frequent oxacillinases involved in resistance of *A. baumannii* strains include OXA-23, OXA-51, and OXA-58 oxacillinases (Sahl et al., 2013), but others such as OXA-21 and OXA-37 type β-lactamases can also occur (Dijkshoorn et al., 2007; Higgins et al., 2010a; Potron et al., 2015). However, none of our isolates were resistant to meropenem. Resistance to carbapenems in *Acinetobacter* can be based on various mechanisms, including the expression of metallo-β-lactamases (class B-β-lactamases) such as MP-1/2/4/5 or VIM 1/2, or class D-β-lactamases OXA-23-like, OXA-24-like, OXA-51-like, or OXA-58 like carbapenemases (Dijkshoorn et al., 2007). OXA-23 mediated resistance to carbapenems is currently the largest concern posed by *A. baumannii* (Feng et al., 2016). The most common carrier of *bla*OXA-23 is ISAba1; this insertion sequence and its transposon vector help insert *bla*OXA-23 into the chromosome and plasmid (Feng et al., 2016). However, none of the food isolates in our study contained the OXA-23-like carbapenemase.

OXA-51-like oxacillinases are chromosomally-encoded, have low level carbapenemase activity and are widely distributed among *A. baumannii* strains (Wallace et al., 2016). Indeed, these could be detected in all strains from food in this study. Although it is typically a weak carbapenemase, acquiring mutations, or moving from the chromosome to a plasmid and thereby increasing the copy number, has been reported to increase activity in some isolates (Wallace et al., 2016). In addition to the *bla*OXA-51-like intrinsic resistance, all strains in this study whose genomes were sequenced showed that they also possessed a *bla*ADC-25 type cephalosporinase that can degrade a variety of cephalosporins such as ceftazidime (Feng et al., 2016). Thirty-six of the *A. baumannii* strains showed resistance to ampicillin by disc diffusion testing. Resistance to aminopenicillins such as ampicillin has been based on *bla*OXA-21-like and *bla*OXA-37-like oxacillinase-mediated resistance, as well as narrow spectrum class A ß-lactamases such as TEM-1- or TEM-2-lactamases (Dijkshoorn et al., 2007). However, none of the strains in our study produced TEM-like beta-lactamases. The results thus showed that *A. baumannii* isolates from powdered milk generally showed resistance to oxacillin of the OXA-51-like type and many also to aminopenicillins, but were mostly susceptible to meropenem. Indeed, genes for carbapenemase involved with meropenem and imipenem resistance such as *bla*OXA-23, *bla*OXA-24, and *bla*OXA-58 could not be detected by PCR. Susceptibility toward carbapenems makes this class of antibiotics the most important to treat clinical infections. Conversely, resistance to these antibiotics dramatically lowers therapeutic options to cure infections (Fishbain and Peleg, 2010).

Most of the strains in this study were intermediate resistant toward the third generation cephalosporin cefotaxime. Cefotaxime resistance is well-known to occur in clinical *A. baumannii* strains (Potron et al., 2015) and has been attributed to the production of CTX-M-2 extended-spectrum class A β-lactamase. It was interesting to observe that this phenotypic (intermediate) resistance was also present in non-clinical strains which stemmed from a dried milk environment in this study. The investigated strains were all susceptible toward the fourth generation cephalosporin antibiotic cefepime.

All isolates were chloramphenicol resistant. A previous study of Roca et al. (2009) reported that most *A. baumannii* isolates were intrinsically resistant to chloramphenicol, and that the mechanism responsible for such resistance was identified as a major facilitator superfamily efflux pump. None of the strains were tobramycin resistant, while about half (44.7%) of the strains were streptomycin resistant. Resistance toward streptomycin and tobramycin was reported to rely on the activities of different enzymes. While tobramycin was reported to rely on modification of the antibiotic mostly by acetyltransferases [(AAC(3)-IIa, AAC(6′)-Ib, AAC(6′)-Ih, AAC(6′)-Iad)], streptomycin was reported to be modified by a nucleotidyl transferase [ANT(3″)-Ia] (Dijkshoorn et al., 2007). The 12 strains for which the complete genome was sequenced in this study all contained a streptomycin 3″-O-adenylyltransferase [(E.C. 2.7.7.47) (AAD(3″))], showing this to be a common resistance gene present in the strains investigated. However, of these 12 strains, only 8 were phenotypically resistant to streptomycin, indicating that in the 4 susceptible strains (43/18, 260/2529, 249/1482, and 64/5920) the AAD(3″) genes may either be non-functional, regulated or that other associated mechanisms for streptomycin such as non-specific pumps were present.

The use of antibiotics in livestock production has been linked to the emergence and spread of resistant bacteria from animals or from foods of animal origin to people (Hamouda et al., 2011). In a previous study, Hamouda et al. (2011) showed that *A. baumannii* strains from a Scottish abattoir showed different epidemiological characteristics to strains isolated from clinics, concluding that the *A. baumannii* isolates from animals were not the precursors of the strains causing hospital infections. The results from our studies show that some *A. baumannii* strains group together with pan-European group II and III clones in groups obtained by rep-PCR genotyping, even though these are not tetracycline resistant and do not stem from a clinical source.

Antibiotic-resistant strains of *A. baumannii/calcoaceticus* complex strains were isolated from infant milk formula in Brazil (Araújo et al., 2015) and were mainly resistant to ampicillin-sulbactam (88.2%) and cefotaxime (82.3%). Similar as in our study, only few strains were resistant to tetracycline (2/17; 11.8%), while 2 and 3 of the 17 strains were resistant to ciprofloxacin (11.8%) and tobramycin (17.6%), respectively (Araújo et al., 2015). These and other studies thus seem to indicate that *A. baumannii* or *A. baumannii/calcoaceticus* complex strains from powdered milk may exhibit single or even multiple antibiotic resistances. In contrast to clinical isolates, however, food isolates are still susceptible to clinically important antibiotics such as tobramycin, meropenem, ciprofloxacin, and cefepime. Indeed our genome comparison data showed that all nine *A. baumannii* food isolates sequenced did not possess chromosomal regions encoding genes for ESBL TEM-1 or high level carbapenemase. One of the main characteristics that accounts for the ability of *A. baumannii* to persist in the clinical setting was its ability to acquire the DNA for numerous antibiotic resistance mechanisms (Roca et al., 2012). Indeed, the acquisition of a multiply drug resistant phenotype was suggested to be a determinant factor for the success of *A. baumannii* as a nosocomial pathogen (Imperi et al., 2011; Antunes et al., 2014). The *A. baumannii* isolates from food, food environments and the associated food route thus seem to hold a relatively lower risk and are thus probably less important in terms of food safety as they are less antibiotic resistant. However, the food route may be a source for contributing new strains or lineages which appear to be inherently able to acquire antibiotic resistance determinants. When such strains enter clinical settings via the food/community route, then could result in the acquisition of new antibiotic resistance genes and the development of new problematic strains.

## Author contributions

All authors listed, have made substantial, direct and intellectual contribution to the work, and approved it for publication.

### Conflict of interest statement

The authors declare that the research was conducted in the absence of any commercial or financial relationships that could be construed as a potential conflict of interest.
